# Explaining deep learning-based representations of resting state functional connectivity data: focusing on interpreting nonlinear patterns in autism spectrum disorder

**DOI:** 10.3389/fpsyt.2024.1397093

**Published:** 2024-05-20

**Authors:** Young-geun Kim, Orren Ravid, Xinyuan Zheng, Yoojean Kim, Yuval Neria, Seonjoo Lee, Xiaofu He, Xi Zhu

**Affiliations:** ^1^ Department of Psychiatry, Columbia University Irving Medical Center, New York, NY, United States; ^2^ Department of Biostatistics, Columbia University Irving Medical Center, New York, NY, United States; ^3^ Mental Health Data Science, New York State Psychiatric Institute, New York, NY, United States

**Keywords:** deep learning, variational autoencoder, resting state fMRI, functional connectivity, autism spectrum disorder

## Abstract

**Background:**

Resting state Functional Magnetic Resonance Imaging fMRI (rs-fMRI) has been used extensively to study brain function in psychiatric disorders, yielding insights into brain organization. However, the high dimensionality of the rs-fMRI data presents significant challenges for data analysis. Variational autoencoders (VAEs), a type of neural network, have been instrumental in extracting low-dimensional latent representations of resting state functional connectivity (rsFC) patterns, thereby addressing the complex nonlinear structure of rs-fMRI data. Despite these advances, interpreting these latent representations remains a challenge. This paper aims to address this gap by developing explainable VAE models and testing their utility using rs-fMRI data in autism spectrum disorder (ASD).

**Methods:**

One-thousand one hundred and fifty participants (601 healthy controls [HC] and 549 patients with ASD) were included in the analysis. RsFC correlation matrices were extracted from the preprocessed rs-fMRI data using the Power atlas, which includes 264 regions of interest (ROIs). Then VAEs were trained in an unsupervised manner. Lastly, we introduce our latent contribution scores to explain the relationship between estimated representations and the original rs-fMRI brain measures.

**Results:**

We quantified the latent contribution scores for both the ASD and HC groups at the network level. We found that both ASD and HC groups share the top network connectivitives contributing to all estimated latent components. For example, latent 0 was driven by rsFC within ventral attention network (VAN) in both the ASD and HC. However, we found significant differences in the latent contribution scores between the ASD and HC groups within the VAN for latent 0 and the sensory/somatomotor network for latent 2.

**Conclusion:**

This study introduced latent contribution scores to interpret nonlinear patterns identified by VAEs. These scores effectively capture changes in each observed rsFC feature as the estimated latent representation changes, enabling an explainable deep learning model that better understands the underlying neural mechanisms of ASD.

## 1 Introduction

The use of functional magnetic resonance imaging (fMRI) data has been pivotal in the field of psychiatry over the last few decades for studying brain function, evaluating underlying neural mechanisms of treatment interventions, and aiding in the diagnosis of a variety of mental disorders. Resting state fMRI (rs-fMRI), which measures spontaneous neural activities in the absence of any specific task, has been useful in revealing intrinsic patterns of brain functional connectivity and providing insights into brain functional organization. One of the most common methods to extract measures of brain networks of rs-fMRI data is to calculate the correlation coefficient between pairs of different regions of interest (ROIs) in the brain. Examining differences in connectivity between healthy controls (HC) and those with psychiatric disorders has helped to understand the underlying neural network deficits associated those disorders (1). In recent years, machine learning approaches have gained prominence for analyzing rs-fMRI data for the diagnosis of psychiatric disorders and the predicting treatment outcomes at the individual level (2).

Despite its utility, leveraging rs-fMRI data presents significant challenges. One of the main chanllenges lies in the high dimensionality of the data. For instance, rs-fMRI data are often partitioned into several hundred ROIs based on various brain atlases (3), e.g., 264 ROIs in the Power atlas (4) or 333 in the Gordon atlas (5). This results in connectivity matrices with more than ten thousand image features, posing substantial challenges for standard machine learning techniques such as random forest, support vector machine, and regressions. Thus, dimensionality reduction methods are often used as a preprocessing step before applying machine learning algorithms to rs-fMRI datasets. Traditional linear methods, such as principal component analysis (PCA) and independent component analysis (ICA), have been used to transform the high-dimensional connectome features into a lower-dimensional space. However, these methods lack to address the complex nonlinear structure of rs-fMRI data. For example, PCA may exhibit biases (6) and ICA can have scalability issues (7) when the data dimension exceeds the sample size. Recently, deep learning-based approaches have extracted low-dimensional latent factors (called *representations*) of resting state functional connectivity patterns (rsFC), showing remarkable performance with expressive nonlinear neural networks (8, 9).

As such, the advent of neural networks has provided new avenues for dimensionality reduction in neuroimaging. One prominent model architecture is the autoencoder (AE) framework (10), which aims to learn a relatively low-dimensional latent representation of the original data, which can then be decoded to recover the data through the decoding phase. Additionally, to produce more effective and interpretable latent representations, variational autoencoder (VAE) approaches have been introduced and have yielded promising results (11). Compared to AEs, VAE approaches possess prominent properties: 1) VAEs are probabilistic models that learn distributions of latent representations, allowing for better modeling of complex data structures. They are seen as an extension of nonlinear ICA (12), using nonlinear neural networks to model the data generation mechanism and creating low-dimensional latent representations consisting of statistically independent components (13). VAEs enable the delineation of each learned component’s role within the framework of nonlinear data generation models, offering a more precise understanding of complex neuroimaging data. In the implementation, the representations follow user-specified distributions called *priors*, and one common choice is multivariate Gaussian distributions. 2) Compared to AEs, the training for VAEs is regularized, which helps prevent overfitting and enforces the independence between components in the estimated representations (14). This makes the representations more interpretable, as each component has a distinct role from all the others (15). For example, in the analysis of hand-written digit images (e.g., Modified National Institute of Standards and Technology [MNIST] http://yann.lecun.com/exdb/mnist/), VAEs may estimate two-dimensional representations where the first component explains the size of digits and the second one explains the slant. In the context of rs-fMRI data, specific latent components may explain within-network or between-network connectivity across various networks. 3) Moreover, VAEs provide personalized inference on the latent space by approximating the distribution of latent representations given the observations. This allows for subject-level information such as uncertainty or variance of estimated representations.

Despite improvements in the performance of machine learning models using latent features, interpreting the significance of the latent features remains a challenge. At first glance, many neural network models can appear as black boxes, with complex and difficult-to-interpret representations. However, strides have been made across the deep learning community to develop tools for interpreting these models. Much of the initial work in visualizing the latent features of autoencoder models originated in the field of computer vision. The tools developed there benefited from the fact that images are relatively easy to interpret and understand naturally by humans. Thus, it was easier to see the qualitative contribution of each latent feature as we could directly examine the images produced by these tools. However, brain imaging modalities such as rs-fMRI do not offer the same level of natural interpretability.

Some initial efforts have been made to generate interpretable latent representations of rs-fMRI data using VAEs. For example, Kim et al. (2021) trained VAEs using large rs-fMRI data from Human Connectome Project. They extracted 2D grids of rs-fMRI patterns at every time as images and input them into the VAE models. The results demonstrated that estimated representations from VAEs effectively characterized individual identification (16). Another study applied VAEs to rs-fMRI data from Autism Brain Imaging Data Exchange (ABIDE) and found an autism spectrum disorder (ASD)-related latent factor (17). However, this study only used a two-dimensional representation space and lacked explanation of the complicated information in rs-fMRI related to ASD. Moreover, the latent representations were based on individual brain regions (e.g., frontal cortices and frontoparietal) rather than brain networks (e.g., executive control network [ECN], salience network [SN], and default-mode network [DMN]).

The goal of this paper is to extract the latent representations from VAE models trained on rs-fMRI data, and create explainable VAE models by visualizing and quantifying the latent representation based on the input rs-fMRI brain features. Here we test the utility of this tool using the rs-fMRI dataset.

## 2 Methods

### 2.1 Dataset

We used publicly available data from the Paris-Saclay Center for Data Science that was initially published for competition in the Imaging-Psychiatry Challenge (IMPAC; https://paris-saclay-cds.github.io/autism_challenge/). The dataset comprised rs-fMRI images from 1,150 participants, including 601 HC and 549 patients with ASD, collected from 35 sites. The demographic characteristics of these participants are detailed in **Table 1**. We further excluded 121 participants who failed to pass quality control procedures.

**Table 1:** The descriptive information for each site from the public dataset.

Site	Total N	Female	Male	Mean Age	StdAge	MinAge	Max Age	Ctrl	ASD	Reject	Accept
0	45	0	45	39.96	14.16	18	62	23	22	6	39
1	32	7	25	7.93	0.97	6	11	15	17	13	19
2	35	11	24	10.43	1.73	8	14	21	14	1	34
3	32	18	14	23.13	11.83	8	47	23	9	5	27
4	17	6	11	26.76	10.01	17	54	11	6	1	16
5	102	38	64	10.43	1.25	8	13	76	26	5	97
6	21	0	21	23.38	3.81	18	33	0	21	0	21
7	48	5	43	10.11	5.98	5	35	16	32	0	48
8	18	3	15	6.69	0.98	5	9	0	18	6	12
9	55	22	33	11.47	2.03	8	15	29	26	1	54
10	32	6	26	13.30	2.97	7	18	13	19	1	31
11	23	0	23	15.43	2.79	12	20	12	11	3	20
12	20	5	15	15.13	1.70	12	18	8	12	2	18
13	20	4	16	22.12	7.67	11	39	10	10	2	18
14	23	6	17	30.86	12.07	18	56	11	12	4	19
15	16	5	11	27.31	6.42	19	40	6	10	3	13
16	32	8	24	10.38	1.31	8	13	18	14	2	30
17	18	0	18	21.89	2.66	18	29	10	8	0	18
18	19	5	14	14.31	1.30	12	17	12	7	3	16
19	40	6	34	28.60	11.76	7	58	26	14	7	33
20	106	25	81	15.52	7.01	7	39	61	45	5	101
21	11	0	11	10.59	1.38	8	13	8	3	0	11
22	24	3	21	17.00	3.57	10	24	12	12	3	21
23	38	6	32	20.03	7.13	9	35	17	21	6	32
25	19	0	19	37.37	8.37	27	64	9	10	7	12
26	23	6	17	14.54	2.01	9	17	15	8	1	22
27	19	3	16	10.13	1.55	8	12	9	10	3	16
28	32	0	32	17.32	3.83	12	26	14	18	2	30
29	38	6	32	12.84	2.23	8	18	18	20	4	34
30	15	1	14	12.42	1.21	10	15	6	9	4	11
31	59	17	42	13.38	2.85	8	19	37	22	9	50
32	28	2	26	15.84	3.60	13	29	16	12	2	26
33	62	0	62	22.10	8.01	9	50	27	35	6	56
34	28	6	22	12.90	2.98	7	18	12	16	4	24
sum	1150	230	920					601	549	121	1029

### 2.2 Image acquisition and processing

All time-series imaging data were acquired using specific atlases and a fetcher provided by IMPAC. We extracted rsFC matrices from the preprocessed rs-fMRI data using Power atlas with 264 ROIs (4). The ROI names and the networks they belong to can be found at https://www.jonathanpower.net/2011-neuron-bigbrain.html. Pairwise correlations were calculated between each ROI for each participant and transformed into a correlation matrix whose elements are Pearson correlations ranging from –1 to 1. Finally, we vectorized correlation matrices by flattening the lower triangular part. For a connectivity matrix with N ROIs, the length of the 1D vectorized correlation vector was calculated by (N−1)×N/2 . The 1D vector was used as an input signal in VAE models. To correct site effects and adjust for age and gender covariates, we performed a Combat algorithm on the correlation vectors using neuroHarmonize (18).

### 2.3 Variational autoencoders

#### 2.3.1 Model architectures

**Figure 1:**
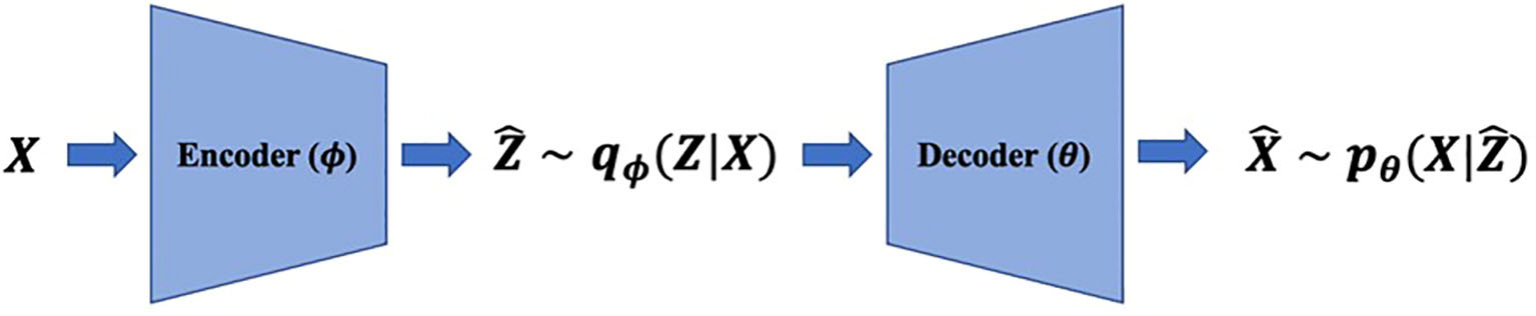
VAEs model the data generation mechanism with low-dimensional representations   and neural networks called decoders. The encoders estimate representations with observations and decoders reconstruct the original data with representations.

We denote high-dimensional observations and low-dimensional representations by $X\in {\mathbb{R}}^{m}$ and $Z\in {\mathbb{R}}^{n}$ , respectively. Realizations of random variables are denoted by small characters. VAEs consist of two parts: (i) encoders modeling the distribution of representations given observations, $q_{\phi }\left(z|x\right)$ , and (ii) decoders modeling the distribution of observations given representations, $p_{\theta }\left(x|z\right)$ (**Figure 1**), where ϕ and θ are neural network parameters. Both $q_{\phi }\left(z|x\right)$ and $p_{\theta }\left(x|z\right)$ are usually modeled as multivariate Gaussian distributions with diagonal variances, $N\left(\mu_{\phi }\left(z|x\right),{\text{Σ}}_{\phi }\left(z|x\right)\right)$ and $N\left(\mu_{\theta }\left(x|z\right),\;I_{m}\right)$ , respectively, to apply reparameterization trick (11) where $I_{m}$ is the identity matrix of size m . Encoders extract representations from observations, decoders reconstruct the original data with them, and they are trained by maximizing evidence lower bounds (ELBOs) (19). For example, for a given x , we can first sample z following $N\left(\mu_{\phi }\left(z|x\right),{\text{Σ}}_{\phi }\left(z|x\right)\right)$ , and then use $\mu_{\theta }\left(x|z\right)$ as reconstruction results.

Compared to AEs, VAEs are distinct in that they are generative models, i.e., they model distributions of observations. Latent representations Z consist of statistically independent components and nonlinear decoders input Z  to model $p_{\theta }\left(x|z\right)$ , e.g., $N\left(\mu_{\theta }\left(x|z\right),\;I_{m}\right)$ . When we assume that the $p_{\theta }\left(x|z\right)$ has zero-variance, the data generation structure of VAE reduces to nonlinear ICA (20). By estimating the nonlinear decoders with likelihood maximization and approximating their inverse mapping with encoders, VAEs can separate blind sources from high-dimensional complicated observations, e.g., functional connectivity.

#### 2.3.2 Loss function

The loss function of VAEs is the negative ELBO, $-E_{q_{\phi }\left(z|x\right)}\log p_{\theta }\left(x|z\right)+D_{KL}\left(q_{\phi }\left(z|x\right)∥p\left(z\right)\right)$ , and is an upper bound of the negative data log-likelihood, $-\log p_{\theta }\left(x\right)$ , where $D_{KL}$ denotes the Kullback-Leibler (KL) Divergence and p(z) denotes a user-specified prior distribution of representations, e.g., multivariate standard Gaussian distributions. Minimizing the loss function of VAEs is equivalent to maximizing a lower bound of likelihoods. In the loss function, the first term is called the reconstruction error which measures how reconstruction results differ from the original observations, and it is the mean squared error when we use Gaussian distributions for decoder distributions. The second term measures the discrepancy between $q_{\phi }\left(z|x\right)$ and p(z) . Considering that the loss function of AEs is the reconstruction error, training VAEs can be viewed as training AEs with the KL-regularization term for the learning data generation mechanism.

### 2.4 Latent contribution scores to explain nonlinear representations

#### 2.4.1 Latent contribution scores

We introduce our latent contribution scores to explain the relationship between estimated representations and the original rs-fMRI brain measures.

For any observation $x\in {\mathbb{R}}^{m}$ , encoder parameter ϕ , and decoder parameter θ , to measure the contribution of each component in $z\in {\mathbb{R}}^{n}$ , we propose a matrix D(x) whose elements are latent contribution scores,


(1) $$D{\left(x\right)}_{kl}=E_{q_{\phi }\left(z❘x\right)}\partial {\hat{x}}_{l}/\partial z_{k},$$

where $\hat{x}$ denotes the reconstruction result, k=1,…,n , and l=1,…,m .

The proposed latent contribution scores in Equation (1) are interpretable in two respects: (i) they are extensions of mixing weights in ICA to the nonlinear data generation mechanism, and (ii) they are input perturbation-based scores (21, 22). For (i), $\hat{x}$ is the reconstruction result with estimated sources whose gradients are mixing weights under a linear generation mechanism. Similar to the explanation on mixing weights in ICA, we can explain the contribution of estimated representations on reconstructions as follows: The increment of the k -th element of estimated representations by one unit yields the increment of the l -th element of reconstructed observations, e.g., the l -th element of reconstructed rs-fMRI functional connectivity, by $D{\left(x\right)}_{kl}$ units on average over $q_{\phi }\left(z❘x\right)$ . With these scores, we can explain how estimated representations change reconstructions in each group, e.g., ASD and HC groups. For (ii), in the interpretable machine learning literature, input perturbation-based scores are types of feature importance measures used to explain how the outputs of complicated and nonlinear networks respond to the perturbation on latent components. This provides an estimate of how important each feature is for the model’s decision-making process. Our scores are input perturbation-based scores in which they average gradients, the marginal changes of outputs by decoders with respect to input (estimated) representations.

#### 2.4.2 Numerical approximation for latent contribution scores

Numerical approximation for the proposed latent contribution scores consists of two parts: (i) approximating gradients for a given representation and (ii) averaging gradients computed in (i) over encoder distributions. For (i), we computed average slopes with small perturbations. Let x be an observation and $\hat{z}$ be an estimated representation sampled from $q_{\phi }\left(z❘x\right)$ . We first compute reconstructions using $\hat{z}$ and $\hat{z}+\varepsilon$ , denoted by $\hat{x}\left(\hat{z}\right)$ and $\hat{x}\left(\hat{z}+e_{k}\right)$ , respectively, where $e_{k}\;$ denotes the k -th component of standard basis of ${\mathbb{R}}^{n}$ , and then compute $\hat{x}\left(\hat{z}+e_{k}\right)-\hat{x}\left(\hat{z}\right)$ which is a numerical approximation of partial gradients up to constant multiplication. For (ii), in computing scores for the k -th latent component, we used fixed points rather than sampling to provide deterministic scores. For all axes except the k -th axis, we used means of encoder distributions, and for the k -th axis, we used pre-specified grid points ranging from means minus three standard deviations to plus three standard deviations. We first approximate gradients at each grid point, and then average them to compute latent contribution scores.

### 2.5 Explaining rs-fMRI and brain networks with latent contribution scores

#### 2.5.1 Model architecture

**Figure 2:**
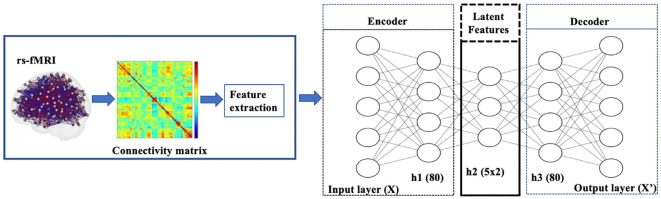
Diagram of VAE pipeline: The model was trained using rs-fMRI data. The samples were then split into a training+validation (70%) and independent-test (30%) data. Then 20% of the training data was set aside for validation and hyperparameter tuning. Once the training+validation was completed, the model’s performance was evaluated on the independent test data, which provides an unbiased estimate of how the model generalizes to unseen data. The resulting VAE model learned to encode patterns from the input brain features into its latent representation.

In our experiments on the rs-fMRI dataset, the observations X are the lower triangular part of functional connectivity matrices from the Power atlas with 264 ROIs. Each element is the correlation of the resting state activity between one of 264 brain regions and another (**Figure 2**). Both the encoder and decoder have one hidden layer. The sizes of the respective layers were chosen by performing a sparse grid search for each of the layers’ sizes independently and evaluating the performance of the model both with respect to the loss function (23). For hyperparameter tuning, we considered the following choices: {tanh, scaled exponential linear unit [SELU]} for activation functions (24); {20, 40, 50, 80, 100, 150, 200, 250} for the number of the hidden nodes; {2, 5, 10, 15, 20} for the latent dimension. We used the loss of VAEs for the model selection criterion. Both the encoder and decoder have one hidden layer. The sizes of the respective layers were chosen by performing a sparse grid search for each of the layers’ sizes independently and evaluating the performance of the model, both with respect to the loss function (23). The chosen number of hidden nodes and latent dimension are 80 and 5, respectively.

#### 2.5.2 Training of VAEs

**Figure 3:**
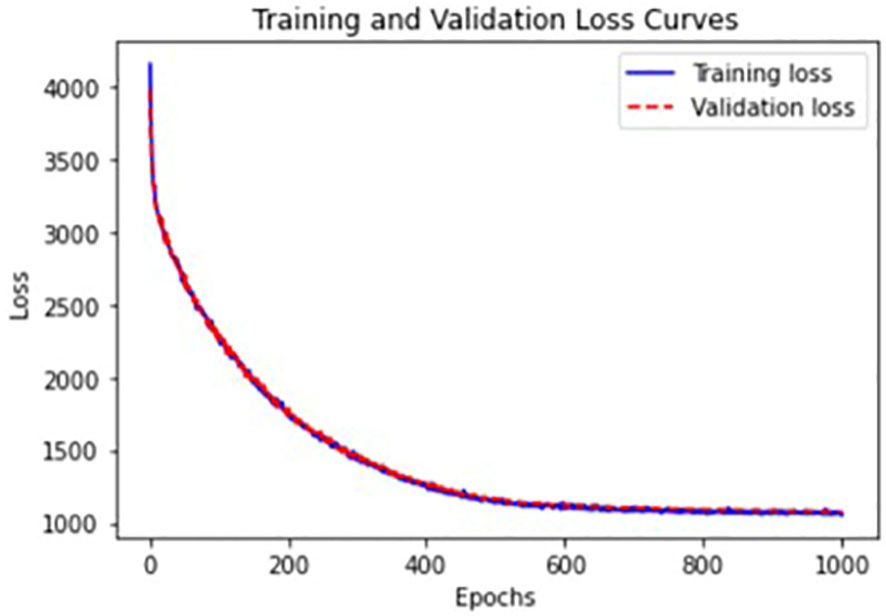
Training and validation loss curves from VAEs with the best validation parameters.

We trained VAEs in an unsupervised fashion without using labels about ASD and HC groups. We standardized the data with median and interquartile ranges and added Gaussian noise with a zero-mean and standard deviation of 0.1 to input data for denoising purposes to learn robust representations (25). The whole dataset was split into training+validation (70%), and test (30%) sets. The batch size, the number of epochs, and the weight decay were 128, 1000, and 0.1, respectively. We applied $L_{2}$ regularization. For the stopping criterion to evaluate convergence, we used the validation loss, negative ELBO. **Figure 3** provides training and validation loss curves. There was no notable overfitting issue.

## 3 Results

**Figure 4:**
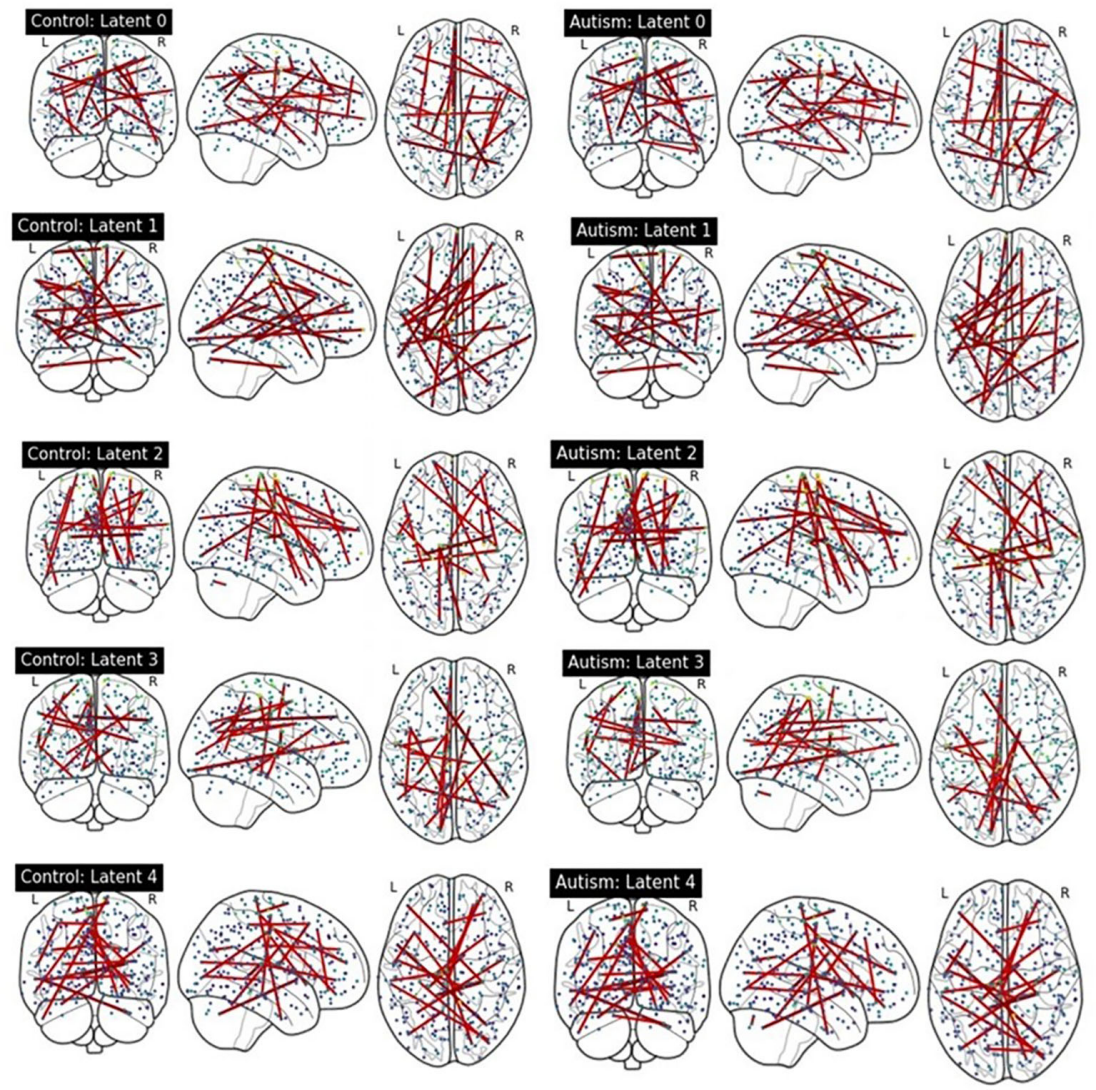
Visualization of top 0.05% functional connectivity based on latent contribution scores. Each row shows results on each component of estimated representations. The left and right columns display results on the ASD and HC groups, respectively.

We compared latent contribution scores of each latent variable based on the rsFC. **Figure 4** provides a visualization of the top 0.05% resting-state functional connectivities with the highest latent contribution scores. The depicted brain network features change the most as the estimated representation (latent) changes.

**Figure 5:**
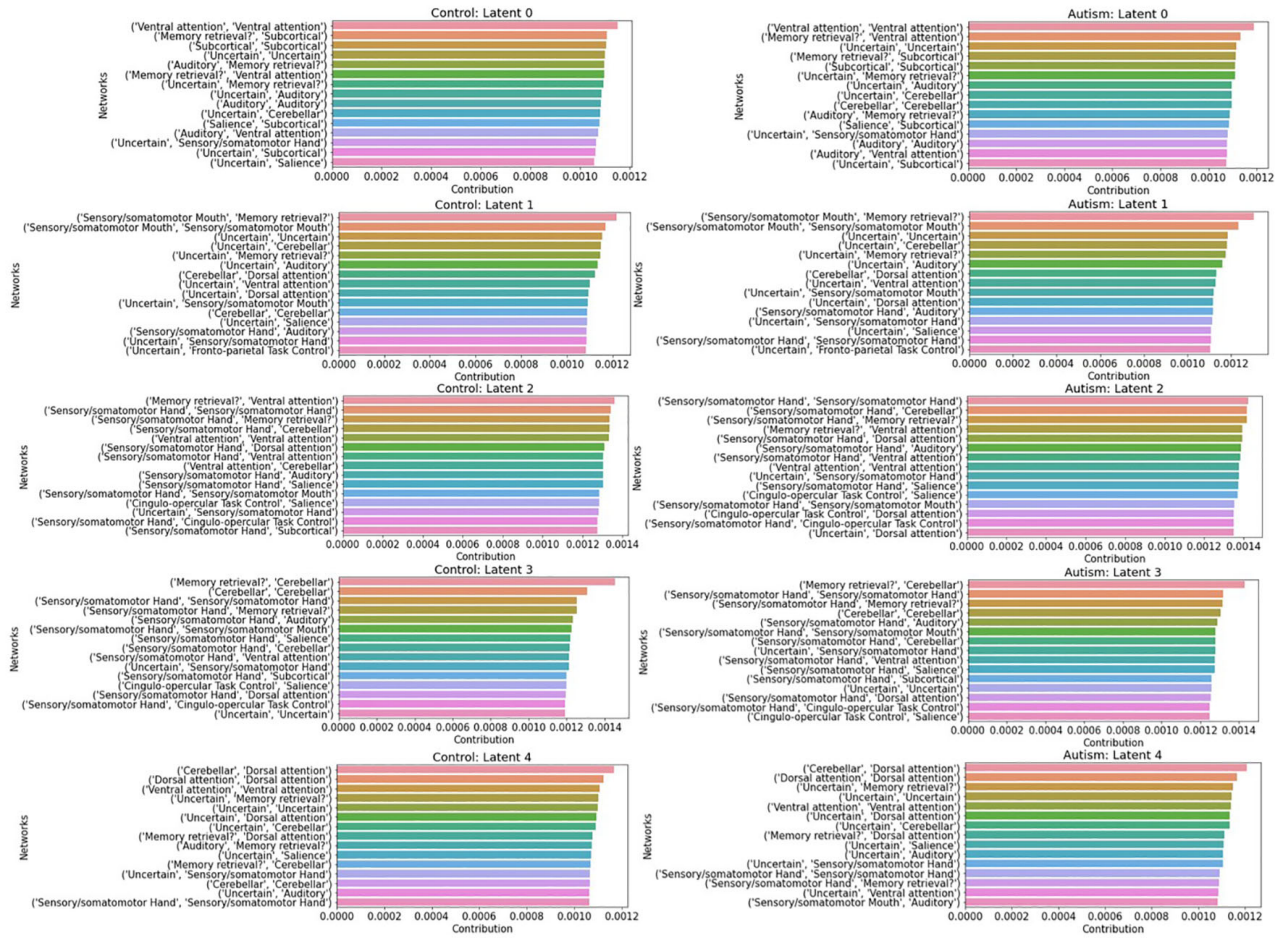
Summary of top 15 network connectivity having the highest latent contribution scores. We averaged scores from rsFC across ROIs over the network level.

We quantified the latent contribution scores for the ASD and HC groups at the network level, as detailed in **Table 2** and **Figure 5**. The ASD and HC groups share the top network connectivity for all estimated latent components. For example, within ventral attention network (VAN) contributes the most to latent 0 in both the ASD and HC. Similarly, latent 1 is primarily influenced by the rsFC between somatomotor (SMN)-memory retrieval networks, latent 2 is driven by the rsFC within SMN; latent 3 is driven by rsFC between memory retrieval-cerebellar networks, and latent 4 is driven by rsFC between cerebellar-dorsal attention networks in both groups.

**Table 2:** Summary of top 3 network connectivity having the highest latent contribution scores.

Latent Representation	Latent contribution score rank	Control Group	Autism Group
Latent 0	Top 1	Within ventral attention	Within ventral attention
	Top 2	Between memory retrieval and subcortical	Between memory retrieval and ventral attention
	Top 3	Within subcortical	Within uncertain
Latent 1	Top 1	Between sensory/somatomotor mouth and memory retrieval	Between sensory/somatomotor mouth and memory retrieval
	Top 2	Within sensory/somatomotor mouth	Within sensory/somatomotor mouth
	Top 3	Within uncertain	Within uncertain
Latent 2	Top 1	Between memory retrieval and ventral attention	Within sensory/somatomotor hand
	Top 2	Within sensory/somatomotor hand	Between sensory/somatomotor hand and cerebellar
	Top 3	Between sensory/somatomotor hand and memory retrieval	Between sensory/somatomotor hand and memory retrieval
Latent 3	Top 1	Between memory retrieval and cerebellar	Between memory retrieval and cerebellar
	Top 2	Within cerebellar	Within sensory/somatomotor hand
	Top 3	Within sensory/somatomotor hand	Between sensory/somatomotor hand and memory retrieval
Latent 4	Top 1	Between cerebellar and dorsal attention	Between cerebellar and dorsal attention
	Top 2	Within dorsal attention	Within dorsal attention
	Top 3	Within ventral attention	Between uncertain and memory retrieval

We averaged scores from rsFC across ROIs over the network level.

Among the rsFC network ROIs that contribute the most to each latent component (as shown in **Table 2**), we further compared them between the ASD and HC groups. We first conducted t-tests to filter ROIs with significantly different latent contribution scores between ASD and HC groups, using a significance level of 1%. Subsequently, we averaged the scores at the network level. We found significant differences in the latent contribution scores in the VAN network (top network driven by latent 0), and the SMN (top 2 network driven by latent 1).

We compared the mutual information (MI) between latent components from Denoising AEs (DAEs) (26) and from our method. DAEs focus solely on minimizing the reconstruction error, aiming for a closer one-to-one relation between the reduced dimensions and the original data, without learning data distributions. The smaller MI indicates weaker dependencies and, consequently, better disentanglement in interpretation. The results showed that our method exhibited a smaller test MI of 0.6253 compared to the DAEs, which had an MI of 0.6511. The DAEs were implemented by removing the KL regularization term from the ELBO.

## 4 Discussion

In this study, we proposed the use of latent contribution scores to explain nonlinear patterns identified by VAEs. These scores effectively capture the marginal changes in each component of the observations as the estimated representations change. With this toolkit, we were able to examine both quantitative and qualitative analyses of the differences in how a VAE-based model represents psychiatric disorders.

Specifically, we were able to quantify which brain networks most significantly contribute to each latent component that differentiated between ASD and HC. We identified five latent components, including within VAN driven latent 0; SMN-memory retrieval networks driven latent 1, memory retrieval-cerebellar networks driven latent 3, and cerebellar-dorsal attention networks driven latent 4. Among these 5 latent components, the latent contribution scores in the ventral attention network driven latent 0, and the SMN driven latent 1 are significantly different between the ASD and HC groups. The VAN and SMN are two important networks implicated in ASD. VAN plays a crucial role in processing sensory information and direct attention. Studies suggest that individuals with ASD have altered rsFC in the VAN, which could contribute to difficulties with focusing, maintaining and shifting attention and social communication (27). The SMN is involved in the processing of sensory information and controlling motor functions. Individuals with ASD often show different sensory processing compared with the HC group. Altered rsFC in the SMN could potentially contribute to these sensory processing differences. Moreover, altered rsFC in SMN may contribute to the motor coordination difficulties in individuals with ASD (28).

Our approach is generally applicable to a broader class of dimension reduction methods, including autoencoders and their derivatives, bidirectional generative adversarial networks (29, 30), and deep belief networks (31) that use probabilistic encoders and any desired imaging modality. In fact, our technique does not require the model to even be a neural network as long as it has mapping from high dimensional observations to estimated representations and vice versa, and the gradient can be numerically approximated. Some examples of models that fall in this category are VAE-based generative adversarial networks (GANs) (32) and hyperspherical VAEs (33). Another advantage of our approach lies in its visualization capability. When observations are visually perceived as in natural images, we can display our latent contribution scores. For example, when the data modality is 4D fMRI voxel-time space data, we can visualize the proposed contribution scores for each latent component in the 4D space and interpret their spatial-temporal patterns.

It is important to note that our analysis was only done on a VAE applied to ROI-to-ROI measures extracted from resting-state timeseries data. Other resting-state measurements such as amplitude of low-frequency fluctuation (ALFF) and regional homogeneity (ReHo) have not been used for this analysis and may be explored in the future works. Additionally, we used the Power atlas (4) with 264 ROIs in this study. Future studies could try leveraging rsFC matrices using different atlases as well. Moreover, as different MRI modalities contain complementary information for ASD, including task-based fMRI, T1 structural MRI and diffusion weighted imaging, fusing multiple modalities may provide additional information, and contribute to each latent component. The proposed deep learning model can potentially be used to combine different imaging modalities via stacked autoencoders, and explain the contributions of each modality to the latent components, which can help in understanding the mechanism of psychiatric disorders such as ASD. The ABIDE dataset primarily focuses on ASD, while ASD frequently coexists with attention deficit hyperactivity disorder (ADHD) and anxiety disorders (34), posing a significant challenge in differentiating the neurodevelopmental impacts of each condition. The comorbidity complicates the analysis, as the overlapping symptoms and neurobiological features may obscure the specific contributions of ASD to brain network configurations. Further research could test the generalizability of our model with respect to comorbidity with other disorders. For example, by comparing latent contribution scores across three groups - HC, ASD without comorbidities, and ASD with comorbidities - we can better dissect the interaction between these disorders and their associations with brain networks.

## 5 Conclusion

In conclusion, our proposed latent contribution scores enhance the interpretability of deep learning models. These models, applied to the rs-fMRI data, can be understood and interpreted by humans. Moreover, explainable VAEs offer insights into which features, from either single modality or a combination of multiple modalities, are most important for particular prediction tasks, such as the classification of ASD from HC. This is valuable for feature engineering and for understanding the underlying neural mechanisms of psychiatric disorders.

## Data availability statement

The original contributions presented in the study are included in the article/supplementary material. Further inquiries can be directed to the corresponding authors.

## Author contributions

YouK: Writing – original draft, Writing – review & editing. OR: Writing – original draft, Writing – review & editing. XYZ: Investigation, Software, Visualization, Writing – review & editing. YooK: Investigation, Writing – review & editing. YN: Investigation, Writing – review & editing. SL: Investigation, Writing – review & editing. XH: Writing – original draft, Writing – review & editing. XZ: Writing – original draft, Writing – review & editing.
